# Rescue surgery for intra-abdominal migration of plastic stents in endoscopic ultrasound-guided hepaticogastrostomy

**DOI:** 10.1055/a-2761-0517

**Published:** 2026-01-08

**Authors:** Masafumi Watanabe, Kosuke Okuwaki, Kazuharu Igarashi, Kai Adachi, Akihiro Tamaki, Yusuke Kumamoto, Chika Kusano

**Affiliations:** 1 Department of Gastroenterolog, Kitasato University School of Medicine, Sagamihara, Japan; 2 38088 Department of General-Pediatric-Hepatobiliary Pancreatic Surgery, Kitasato University School of Medicine, Sagamihara, Japan

Plastic stents (PSs; Type IT, Gadelius Medical K.K., Tokyo, Japan), designed specifically for endoscopic ultrasound-guided hepaticogastrostomy (EUS-HGS), feature a pigtail shape on the gastric side, and migration into the abdominal cavity is particularly rare 1 2 . We experienced a case of intra-abdominal migration of a dedicated PS during EUS-HGS and successfully performed biliary drainage by returning the migrated PS to the stomach through emergency surgery.

**Fig. 1:**
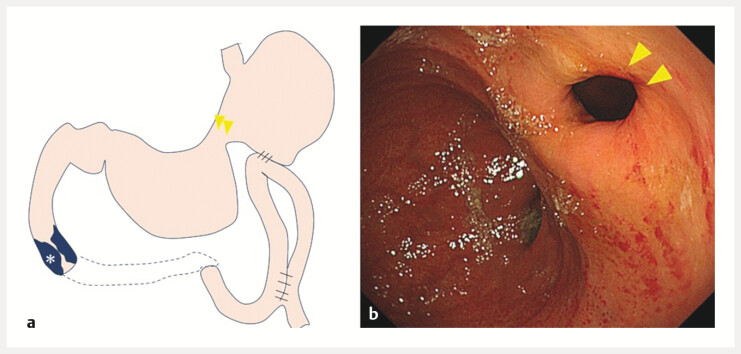
Stomach-partitioning gastrojejunostomy. **a** Schematic illustration showing suturing of the lesser curvature of the gastric body (yellow arrowhead). The tumor is causing narrowing of the duodenum (*). **b** An endoscopic image demonstrating the same finding, with the lesser curvature of the gastric body sutured (yellow arrowhead).

The patient was a 91-year-old man, who had previously undergone stomach-partitioning gastrojejunostomy ( Fig. 1 3 ) for duodenal cancer. EUS-HGS was performed to treat obstructive jaundice. Due to the suturing of lesser curvature, endoscopic visualization was not possible. Therefore, the PS was deployed under fluoroscopic guidance. The stent was not visible in the stomach, suggesting intra-abdominal migration. Computed tomography confirmed that the tip remained in the bile duct, while the gastric side had entered the abdominal cavity. Emergency surgery was performed. The migrated stent was confirmed intraoperatively. The original puncture site had closed naturally, a new fistula site was thus created at the position where the PS was stretched straight. A purse-string suture (3–0 Vicryl, Ethicon, Inc., New Jersey, USA) was placed, and the stomach was opened using mosquito forceps. The stent was stabilized at the hepatic side and repositioned into the stomach, ligated, and fixed. To promote fistula formation, the gastric serosal muscle and liver parenchyma were sutured. Surgery was completed within 60 min, followed by intra-abdominal lavage and drain placement ( Video 1 ).

Rescue surgery was performed for intra-abdominal migration of plastic stents during endoscopic ultrasound-guided hepaticogastrostomy.Video 1

Obstructive jaundice resolved, and the patient was discharged on postoperative day 11. Although biliary obstruction recurred 49 days later, endoscopic stent exchange via the fistula was successful.

In conclusion, when a PS migrates into the abdominal cavity during EUS-HGS and its tip remains in the bile duct, surgical repositioning could correct the migration stent and allow for biliary drainage. Surgery was completed within 60 minutes. Obstructive jaundice resolved, and the patient was discharged on postoperative day 11.

Endoscopy_UCTN_Code_CPL_1AL_2AD
